# Fertility desire and associated factors among reproductive age women on anti-retroviral therapy in East Wollega public health facilities, west Ethiopia: facility based cross-sectional study

**DOI:** 10.3389/frph.2025.1449392

**Published:** 2025-06-12

**Authors:** Worku Fikadu

**Affiliations:** Department of Public Health, Institute of Health Science, Wollega University, Nekemt, Ethiopia

**Keywords:** PLHIV, fertility desire, reproductive women, East Wollega, Ethiopia

## Abstract

**Background:**

Fertility desire in people living with HIV is the desire of people to have children in the future despite a diagnosis of HIV. The desire to have children among reproductive-age women living with HIV has signiﬁcant implications for the transmission of HIV to sexual partners and newborns in the future. There is no study conducted to determine the magnitude of the fertility desire among women of reproductive age living with HIV in East Wollega western Ethiopia. Therefore this study aimed to determine fertility desire and associated factors among women of reproductive age living with HIV receiving ART.

**Objectives:**

To assess fertility desire and associated factors among reproductive-aged women on antiretroviral treatment in East Wollega, West Ethiopia, 2023.

**Methods:**

A facility-based cross-sectional study was conducted in East Wollega from January 01/2023 to 30/2023. Data was collected from 419 study participants using interviewer-administered structured questionnaires by employing systematic random sampling techniques. Data entry and analysis were performed using Epi Data version 3.1 and SPSS version 23, respectively. Both bivariate and multivariate analyses were carried out using binary logistic regression. Statistical significance was established for variables with *P* < 0.05 in the multivariate analysis.

**Results:**

Among the 419 respondents, 166 (39.62%) reported that they desired to have children in the future. Age between 18 and 24 years (AOR = 2.49, 95% CI; 1.16, 5.35), having partner Living with HIV: (AOR = 0.50, 95% CI; 0.25, 0.9), and being married (AOR = 2.69, 95% CI; 1.65, 4.39) were significantly associated with fertility desire.

**Conclusion:**

The proportion of women of reproductive age living with HIV who desired children was high in this cohort. Understanding the fertility desire among women living with HIV/AIDS has a significant role in reducing perinatal transmission of HIV. Hence, Health care workers should provide intensive counselling regarding the possibility of the mother-to-child transmission of HIV.

## Introduction

Fertility is the ability conceive a child through regular sexual activity. The desire for fertility for people living with HIV is the desire of people to have children in the future despite having a diagnosis of human immunodeficiency virus (HIV) (1–4).

Women living with HIV have decreased desire for fertility due to various opportunistic infections. HIV infection results in decreased spermatozoa production, coital frequency, fetal survival, and partner survival (2, 5–7). According to community perception People living with HIV (PLWH) and related opportunistic infections cannot be sexually active, marry, or commit to divorce (3, 6). Community norms, stigma, and discrimination also influence the desire to have children (8, 9).

HIV can be transmitted from mother to child throughout the antepartum (5%–10%), intrapartum (10%–15%), and postpartum (30%–45%) periods, depending on the timing of infection and the availability of services for prevention of mother-to-child transmission (PMTCT) (4). PMTCT is the main pillar in the reduction of perinatal transmission to less than 2% among the non-breastfeeding population and 5% among the breastfeeding population (2). The aim of the PMTCT program in Ethiopia was to achieve 95% ART coverage for pregnant women living with HIV by 2015 (5).

A 4-pronged approach toward preventing HIV transmission includes preventing unintended pregnancy, preventing HIV transmission from mothers living with HIV to their infants, and providing treatment and support for women infected with HIV, their infants, and their families (4, 5). Couples have the right to marry, express sexuality, and experience parenthood, regardless of their HIV status (2, 8). Fertility desire among women living with HIV ranges from 12.1% in Asian countries to 50.8% in sub-Saharan African countries. In Ethiopia, fertility desire ranges from 34% to 45% (9–11). However, these women face challenges such as high HIV prevalence, poor health systems, and the risk of MTCT (5, 12–14). Decreased fertility desire is influenced by fear of perinatal HIV transmission, preterm delivery, divorce, HIV-related stigma, and poor health status (15–17). Low PMTCT coverage and poor integration with maternal services are the main challenges faced by the Ethiopian government. To address these issues, Ethiopia has adopted the WHO PMTCT strategy, which has increased fertility desire due to improvements in quality of life, survival rate, and reproductive health services (2). There is no study conducted to determine the magnitude of the fertility desire among women of reproductive age living with HIV in east Wollega western Ethiopia. Hence; this study assessed fertility desire among women of reproductive age living with HIV in East Wollega, Ethiopia.

## Methods and materials

### Study setting and period

The study was conducted in two public hospitals and six health centers in the eastern Wollega zone. The East Wollega Zone is located 333 km from the capital city of Addis Ababa in western Ethiopia. According to the 2007 census, this zone has a total population of 1,213,503 people, of whom 606,379 are men and 607,124 are women. There were 16,623 people currently on ART in the study area, 5,385 of whom were reproductive-aged women. Of the 5,385 reproductive-aged women, 2,311 were receiving ART services at eight randomly selected public health facilities. This study was conducted from January 1, 2023, to January 30, 2023.

### Study design

A facility-based cross-sectional study was conducted.

### Study population

All reproductive-aged women who were receiving ART during the study period at selected public health facilities in the east Wollega zone.

### Inclusion and exclusion criteria

The study enrolled women living with HIV who had a follow-up and lived longer than 6 months. We exclude all women who were critically ill and those who had a hearing problem.

### Sample size determination and sampling procedure

**Figure 1:**
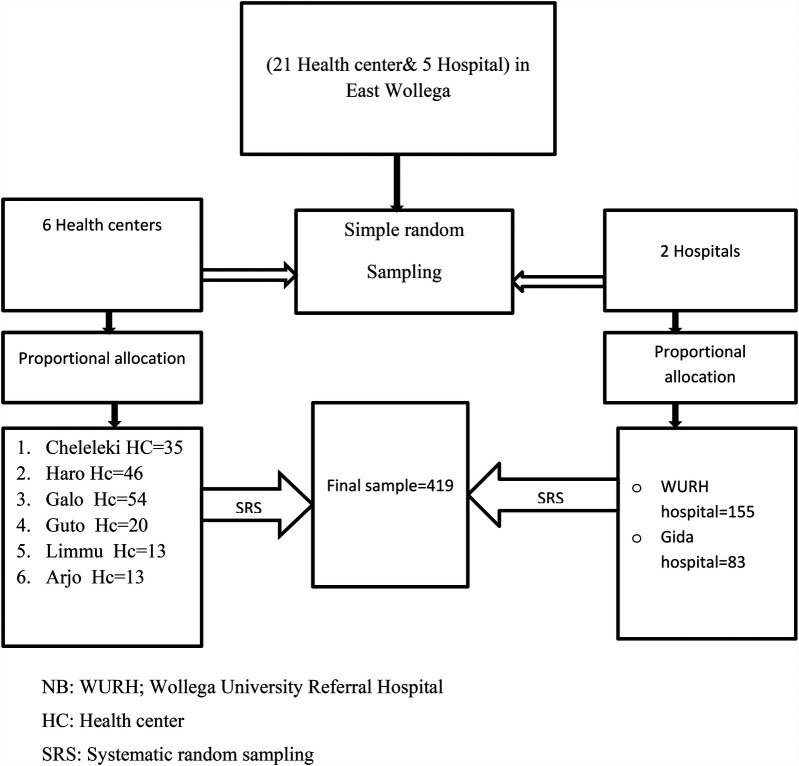
Schematic representation of the sampling procedures, showing the sampling procedure for reproductive-aged women on an ART in public health facilities in the east Wollega zone, Oromia, Ethiopia 2023. NB, WURH; Wollega University Referral Hospital; HC, health center; SRS, systematic random sampling.

The single population proportion formula (P) was used to determine the sample size by considering the following assumptions. Based on the findings from Addis Ababa, the prevalence of fertility desire was 54.6% (10), with a 95% confidence level and a 5% margin of error (d). Finally, with the addition of the 10% nonresponse rate, the final sample size became 419. The total sample size was proportionally allocated to the 8 randomly selected ART-providing public health facilities, and a systematic random sampling technique was employed to recruit the study participants. The first participant was randomly selected, and the remainder of the study unit was recruited based on the calculated k^th^ (K = 5) interval until the required final sample size was achieved (Figure 1).

### Study variables

#### Dependent variable

Fertility desire (Yes, No).

The independent variables included sociodemographic factors (age, educational level, marital status, occupational status), sex-related factors, family planning, reproductive-related factors, clinical factors related to HIV, partner-related factors, knowledge about PMTCT and MTCT, and community pressure.

#### Fertility desire

A psychological state in which someone has the personal motivation to have a child. Those who are motivated to have more children in the future have fertility desire. Those who have no motivation to have more children have no fertility desire (10).

#### Treatment stage

This stage is based on the WHO classification of a patient's clinical condition on ART.

The viral load is the number of RNA copies per milliliter of blood (18).

#### Regular partner

Women who had regular sexual relationships (19) with men.

#### Sexually active

Women who have performed at least one sexual practice during the last six months (20).

#### Community pressure

Women who have community approval/disapproval for having children irrespective of their HIV status. If there is intense pressure from family, spouses and friends to reproduce, this pressure is considered yes, and the reverse is true (5).

### Data collection methods and instruments

The data were collected using pretested, interviewer-administered structured questionnaires. The questionnaire was initially prepared in English and then translated into the Afaan Oromo language and returned to English to check for validity and reliability. Data collectors and supervisors were recruited from health professionals in the respective health facilities, and 3 days of training was given prior to data collection on the methods of data handling and supervision accuracy.

### Data quality control

The prepared tool was pretested on 5% of the population in the Nekemt Specialist Hospital. The results of the pretest were analyzed, and necessary modifications were made. Three days of training were provided to ensure data quality. The completed questionnaires were cross-checked by supervisors and the principal investigator to ensure data completeness. Data cleaning and checking for missing values were conducted prior to analysis.

### Data processing and analysis

The data were entered into EPI Data Version 3.1 and exported to SPSS Version 22.0 for analysis. Descriptive statistics were used to summarise the frequency, percentage, and mean of the characteristics of the study participants. Multicollinearity was assessed by the variance inflation factor (VIF) >10, which was considered problematic. Model fitness was checked by Hosmer and Lemshow's goodness-of-fit test. Binary logistic regression analysis was used to ascertain the associations between explanatory variables and outcomes. Variables with a *P*-value less than 0.25 in the bivariable analysis were entered into the multivariable analysis. Variables with *p* < 0.05 in the multivariable logistic regression were considered to have a statistically significant association with the outcome variable.

## Results

### Sociodemographic characteristics of the study respondents

A total of 419 respondents, participated in the study with a response rate of 100%. The mean age of the respondents was 32.8 years (SD ± 6.92), and the majority of the respondents were 24–34 years old, accounting for 194 (46.30%) of the sample. Approximately 318 (75.89%) of the reproductive-age women who participated in the study lived in urban areas. The majority of the study subjects (238, 56.94%) were orthodox Christians and 218 (52.08) were housewives (Table 1).

**Table 1:** Clinic in east Wollega public health facilities in western Ethiopia, 2023.

Variables (factors)	Category	Frequency	Percent
Age	18–24	58	13.84
	25–34	194	46.30
	>35	167	39.86
Residence	Urban	318	75.89
	Rural	101	24.11
Religion	Orthodox	238	56.94
	Muslim	151	36.12
	Protestant	24	5.74
	Catholic	5	1.20
Marital status	Single	59	14.08
	Married	248	59.19
	Widowed	48	11.46
	Divorced	64	15.27
Educational status	Can't read and write	127	30.31
	Can read and write	138	32.94
	Primary	58	13.84
	Secondary	50	11.93
	College and above	46	10.98
Occupational status	Housewife	218	52.03
	merchant	67	15.99
	Gov't employed	59	14.08
	Non gov't employ	53	12.65
	Farmer	17	4.06
	Others	5	1.19

### Reproductive history, family planning, sex-related factors, and reasons for fertility desire

Regarding the desire to have children, approximately 166 (39.62%) respondents said they had a desire for fertility or more children. Seventy-eight percent of the study participants got pregnant at least once during the data collection period among those, 180 (55.02%), became pregnant after receiving an HIV diagnosis. Of the study study subjects, 109 (26.02%) had three or more children. Approximately 215 (64.95%) individuals used contraceptives, 76 (35.35%) of whom used an injectable form of birth control. Of the study subjects 274 (65.39%) disclosed their HIV status to their sexual partners. The time for future child desire was not decided in 65 (39.16%) participants, and approximately 23% of people want to have a child within two to three years. Approximately 154 (36.75%) had community pressures that influenced them to have children (Table 2).

**Table 2:** Reproductive history, family planning, sexually related factors and reasons for fertility desire among HIV-positive reproductive age women attending ART clinics in east Wollega public health facilities, western Ethiopia, 2023 (*n* = 419).

Variables	Categories	Frequency	Percentage
Gravidity history	Null	92	21.96
	Primigravida	111	26.49
	Multi gravida	216	51.15
Abortion history	No	301	92.1
	Yes	26	7.9
No living children	0 (no child)	118	28.16
	1–2	192	45.82
	3–4	92	21.96
	≥5	17	4.06
history of having died child	No	298	91.13
	Yes	29	8.87
Current pregnant status	No	331	79.00
	Yes	88	21.00
Current contraceptive utilization	No	116	35.05
	Yes	215	64.95
Types of contraceptive	Injection	76	35.35
	Implant	62	28.84
	Pills	40	18.60
	Others	37	17.2
Having sexual activity in the last 6 months	No	70	16.71
	Yes	349	83.29
No sexual partners in the last 12 months	1	302	72.08
	>2	117	27.92
Do you have a regular partner	No	154	36.75
	Yes	265	63.25
If yes, did you disclose HIV status to your partner?	No	145	34.61
	Yes	274	65.39
Fertility desire	No	253	60.38
	Yes	166	39.62
Times to desire future	1year	25	15.06
	2–3 year	39	23.49
	After 3 year	37	22.29
	Not decided	65	39.16
No of the child want for future	1	12	7.23
	2	62	37.35
	>3	46	27.71
	I don't know	46	27.71
Reason for future child	No child before	50	30.12
	Importance of parenting	32	19.28
	Partner desire	34	20.48
	Can have an HIV-negative child	50	30.12
Does the community have pressure for having children?	No	265	63.25
	Yes	154	36.75
Sex preference	No	118	71.08
	Female	25	52.08
	Male	23	47.92

### Clinical characteristics and knowledge of MTCT and PMTCT

Of the study subjects, 125 (29.83%) and 294 (70.17%) individuals had been on ART for 5 or fewer years and more than 5 years, respectively. Of the total participants 44 (10.5%), 79 (18.85%), and 296 (70.64%) had CD4 counts less than or equal to 200, 201–499, and 500 and greater per microliter of blood, respectively. Of the total participants in the study, 195 (78.31%) responded that their partners' HIV status was positive (Table 3).

**Table 3:** Clinical characteristics and knowledge-related factors MTCT and PMTCT among HIV-positive reproductive age women attending ART clinics in east Wollega public health facilities in western Ethiopia, 2023 (*n* = 419).

Variable	Categories	Frequency	Percentage
Duration on ART	<5	125	29.83
	>5	294	70.17
Regimen type	1st line	372	88.78
	2nd line	47	11.22
HIV status of your partner	Negative	54	21.69
	Positive	195	78.31
Perceived current health status	Improved	304	72.55
	Same	77	18.38
	Deteriorated	38	9.07
Recent CD4 count indicates	<200	44	10.50
	200–499	79	18.85
	≥500	296	70.64
Recent Viral Load indicates	<1,000 copies	356	84.96
	>1,000 copies	63	15.04
Discussed jointly with partner about pregnancy, MTC …	No	259	61.81
	Yes	160	38.19
Do you know about MTCT?	No	113	26.97
	Yes	306	73.03
Do you know about PMTCT?	No	53	12.65
	Yes	366	87.35

### Factors associated with fertility desire

The following variables were significantly associated with fertility desire in this study population: age, marital status, having a regular partner, educational status, community pressure, number of living children, and HIV status of the partner. The odds of fertility desire were 2.5 times greater among women aged 18–24 years than among those aged ≥35 years [AOR = 2.5, *p* = 0.019, 95% CI (1.16, 5.35)]. The odds of having fertility desire were 2.69 times greater among married women than among unmarried women [AOR = 2.69, *p* = 0.000*, 95% CI (1.65, 4.39)]. Study participants who had a college education or above had 3.02 times greater fertility desire than those who could not read or write [AOR = 3.02, *p* = 0.008, 95% CI (1.3, 6.84)]. The odds of fertility desire were 2.72 times greater and 3.72 times greater among mothers who had no or less than 2 children, respectively, than among mothers who had more than three children [AOR = 2.72, 95% CI (1.35, 5.5)] and [AOR = 3.72, 95% CI (2.0, 6.8)], respectively (Table 4).

**Table 4:** Bivariable and multivariable logistic regression analysis of factors associated with fertility desire among PLHIV of reproductive age in east Wollega public health facilities, 2023.

Variables	Categories	Fertility Desire		COR 95% CI	AOR 95% CI	*p*-value
		Yes	No			
Age	18–24	34	24	3.1 (1.69, 5.8)	2.5 (1.16, 5.35)	0.019
	24–34	80	114	1.55 (1.01, 2.99)	1.5 (.91, 2.57)	0.102
	>35	52	115	1	1	
Educational status of the respondent	Can't read &write	44	83	1	1	
	Read & write	54	84	1.21 (0.73, 2.00)	1.23 (0.75, 2.37)	0.467
	Primary	18	40	0.85 (0.43, 1.65)	0.65 (0.29, 1.45)	0.300
	Secondary	19	31	1.15 (0.59, 2.27)	1.21 (0.56,2.58)	0.425
	Collage & above	31	15	3.89 (1.90, 7.98)	3.02 (1.3, 6.84)	0.008
Marital status	Un married	56	115	1	1	
	Married	110	138	1.63 (1.09, 2.45)	2.69 (1.65, 4.39)	0.000*
Number of living children	No child	76	42	1.76 (0.98, 3.15)	2.72 (1.35, 5.5)	0.000*
	1–2	98	94	3.32 (1.97, 5.61)	3.72 (2.0, 6.8)	0.005
	≥3	26	83	1	1	
Partner HIV status	Negative	30	24	1	1	
	Positive	136	229	0.47 (0.266, 0.84)	0.50 (0.25, 0.99)	0.005
Duration on ART	<5	42	83	1	1	
	≥5	124	170	1.44 (0.93, 2.23)	1.41 (0.83,2.36)	0.197
Treatment type	1st line	154	218	1	1	
	2nd line	12	35	0.48 (0.24, 0.96)	0.46 (0.20, 1.05)	0.068
Having regular partner	No	44	110	1	1	
	Yes	122	143	2.13 (1.39, 3.26)	1.73 (1.01, 2.97)	0.045
Disclose HIV status	No	50	95	1	1	
	Yes	116	158	1.39 (0.91, 2.11)	0.96 (0.57, 1.62)	0.891
Community pressure	No	112	153	1	1	
	Yes	54	100	0.73 (0.48, 1.11)	0.45 (0.27, 0.75)	0.002
Number of sexual partner	One	110	192	1	1	
	Two or more	56	61	1.6 (1.0, 2.46)	1.37 (0.79, 2.36)	0.256
Vaginal discharge	No	137	232	1	1	
	Yes	29	21	2.3 (1.2, 4.2)	1.41 (0.68, 2.92)	0.344

## Discussion

The findings of this study showed that 39.6% of the study subjects desire to conceive. This finding was in line with a study conducted in Tanzania and Uganda which ranges from 35% to 39.5 (21–23). However, this percentage was lower than that reported in studies in Addis Ababa (54.6%) (10), Harrison (52.9%) (24), and South Africa (80%) (25). This disparity might be due to differences in the study period, study design, study area, and population under study.

Factors associated with the desire to conceive included being married or having a regular partner (AOR 2.69), having a college education (AOR 3.02), and having no children (AOR 2.72). The odds of having a fertility desire were 2.69 times greater among women who were married or had regular partners compared to those who were unmarried or had irregular partners of reproductive age living with HIV. These findings align with research from India (26), northern Nigeria (27, 28), and Brazil (29). This may be attributed to the fact that being in a committed relationship provides a sense of security and reliable support for raising children. Furthermore, the odds of having a fertility desire among women with regular partners were also 2.69 times greater than those among women with irregular partners, consistent with findings from Addis Ababa (10), Hawasa (8), and Jimma (18). This is due to a greater ability to make shared decisions between the partners on having children who are free from human immune deficiency virus.

Study participants who had a college education or above had 3.02 times greater fertility desire than those who could not read or write. This finding is supported by a study at the Amhara Regional Referral Hospital, which showed that having no formal education decreased fertility desire by 49% (1). This might be attributed to the fact that educated people have better decision-making skills than individuals with no formal education. Further, more educated people have a better understanding of mother to child transmission and available prevention services.

Having a regular partner increases the odds of fertility desire among PLHIV by 2 times compared to those who do not have a regular partner, and having a partner living with HIV decreases fertility desire by 50%. These findings were supported by similar studies in India, Nigeria, and Brazil (26–29). However, this finding contradicts findings from a study conducted in Australia (3, 19). This might be due to greater perceived stigma against their children in the community and lower economic capability for caring for their children whenever both couples are living with HIV.

The odds of fertility desire were 2.72 times greater and 3.72 times greater among mothers who had no children and who had ≤2 children, respectively, than among mothers who had more than three children**.** This finding is consistent with studies from northern Nigeria (28), Brazil (29), and Uganda (22). This might be attributed to the intensive counseling they received during each delivery at health facilities on PMTCT and reproductive health services and their greater awareness of further pregnancy. However, this finding contradicts findings from a study in Harari. This might be due to the lower PMTCT service and lower engagement in decision making regarding the number of children in this area compared to the current study setting (24).

### Limitations of the study

Due to the nature of cross-sectional study design, establishing cause-and-effect relationships is difficult.

## Conclusion

In this study, the magnitude of desire for fertility among reproductive-aged women living with HIV was high. Age, marital status, level of education, number of children still alive, having a regular partner, community pressure, and partner HIV status were found to be factors associated with the desire for reproduction among PLHIV.

## Recommendation

Based on the findings of the study, the following recommendations have been proposed.

## For health care providers, health care facilities, zonal health departments, regional health bureaus and ministries of health

⮚ Health care providers should provide intensive counselling regarding the possibility of the mother to child transmission of HIV. Health care providers should strengthen counseling on MTCT for Women who had no child.
⮚ Health professionals should strengthen the involvement and support of their partner during antenatal care visits and on antiretroviral drug adherence to minimize the risk of HIV transmission.
⮚ The regional health bureau and Zonal Health Department should emphasize the integration of family planning with HIV/AIDS care for women of reproductive age living with HIV to prevent unwanted pregnancy.
⮚ The Ministry of Health should increase services and support for MTCT and PMTCT.

## Data Availability

The original contributions presented in the study are included in the article/Supplementary Material, further inquiries can be directed to the corresponding author.
